# Mining of Opinions on COVID-19 Large-Scale Social Restrictions in Indonesia: Public Sentiment and Emotion Analysis on Online Media

**DOI:** 10.2196/28249

**Published:** 2021-08-09

**Authors:** Andi Muhammad Tri Sakti, Emma Mohamad, Arina Anis Azlan

**Affiliations:** 1 Centre for Research in Media and Communication Faculty of Social Sciences and Humanities Universiti Kebangsaan Malaysia Bangi Malaysia; 2 UKM x UNICEF Communication for Development Centre in Health Faculty of Social Sciences and Humanities Universiti Kebangsaan Malaysia Bangi Malaysia

**Keywords:** large-scale social restrictions, social media, public sentiment, Twitter, COVID-19, infodemiology, infoveillance

## Abstract

**Background:**

One of the successful measures to curb COVID-19 spread in large populations is the implementation of a movement restriction order. Globally, it was observed that countries implementing strict movement control were more successful in controlling the spread of the virus as compared with those with less stringent measures. Society’s adherence to the movement control order has helped expedite the process to flatten the pandemic curve as seen in countries such as China and Malaysia. At the same time, there are countries facing challenges with society’s nonconformity toward movement restriction orders due to various claims such as human rights violations as well as sociocultural and economic issues. In Indonesia, society’s adherence to its large-scale social restrictions (LSSRs) order is also a challenge to achieve. Indonesia is regarded as among the worst in Southeast Asian countries in terms of managing the spread of COVID-19. It is proven by the increased number of daily confirmed cases and the total number of deaths, which was more than 6.21% (1351/21,745) of total active cases as of May 2020.

**Objective:**

The aim of this study was to explore public sentiments and emotions toward the LSSR and identify issues, fear, and reluctance to observe this restriction among the Indonesian public.

**Methods:**

This study adopts a sentiment analysis method with a supervised machine learning approach on COVID-19-related posts on selected media platforms (Twitter, Facebook, Instagram, and YouTube). The analysis was also performed on COVID-19-related news contained in more than 500 online news platforms recognized by the Indonesian Press Council. Social media posts and news originating from Indonesian online media between March 31 and May 31, 2020, were analyzed. Emotion analysis on Twitter platform was also performed to identify collective public emotions toward the LSSR.

**Results:**

The study found that positive sentiment surpasses other sentiment categories by 51.84% (n=1,002,947) of the total data (N=1,934,596) collected via the search engine. Negative sentiment was recorded at 35.51% (686,892/1,934,596) and neutral sentiment at 12.65% (244,757/1,934,596). The analysis of Twitter posts also showed that the majority of public have the emotion of “trust” toward the LSSR.

**Conclusions:**

Public sentiment toward the LSSR appeared to be positive despite doubts on government consistency in executing the LSSR. The emotion analysis also concluded that the majority of people believe in LSSR as the best method to break the chain of COVID-19 transmission. Overall, Indonesians showed trust and expressed hope toward the government’s ability to manage this current global health crisis and win against COVID-19.

## Introduction

Social quarantine measures, such as movement control order, shelter in place, and lockdowns, are considered effective methods to curb the spread of COVID-19 [1]. Countries all over the globe affected by this pandemic have at least considered implementing this modus as a national strategy to bring COVID-19 cases down and reduce the burden on their health care system. Until a vaccine can be effectively administered to the public, the only effort that can be done to reduce virus transmission sustainably is by exercising prevention measures and embracing it as the new living norm. This includes restricting movement that reduces social contact and improves physical distancing [2]. Physical distancing is performed to decrease the risk of virus transmission from one who is likely to be infected to other healthy individuals [3]. A study measuring the effectiveness of physical distancing in England found that this method can significantly decrease the contact-level frequency up to 74% and was the key factor in decreasing the total confirmed cases in the country [4]. Effective physical distancing correlates with a gradual decline in the number of cases infected [5].

In most countries affected by COVID-19, national-level physical distancing order such as lockdowns, movement control, and shelter in place are implemented as strategies to bring down the number of coronavirus cases. This regulation has been proven as the most effective strategy to eradicate the spread of the virus in several countries such as Brunei [6], New Zealand, and Vietnam [7]. As a result, those 3 countries have succeeded in flattening the curve since the first wave of infection up until now. A valuable lesson can also be learned from Indonesia’s closest neighboring country—Malaysia. Through a study on COVID-19 control measures, Ng et al [8] summarized that the movement control order had managed to suppress the transmission within only 3 weeks after its implementation. Thus, despite difficulties in implementing physical distancing measures in the long run, this method is a critical and effective measure in curbing the spread of infection [9].

After the first case was reported on March 2, 2020, the COVID-19 curve of Indonesia started to show a significant increase at the end of the month with no signs of decline, as the number of infected cases continued to hit a new peak every day. In that month, with 136 deaths out of 1528 total positive cases, Indonesia surpassed China in its case fatality rate (8.90%). China’s death rate was at 4% at the time [10]. The implementation of the large-scale social restriction (LSSR) on March 31, 2020, was overdue, as the number of cases was significantly high [11]. At the time, the number of positive cases in Indonesia was still 2 times lower compared with Malaysia, but as of early June 2020, the country had the second highest number of positive confirmed cases in Southeast Asia after Singapore. Indonesia became the country with the highest fatality rate in Asia, with a case fatality rate of 5.94% (n=1573) among the overall confirmed cases (N=26,473) [12]. If social restriction is a reference to the success of a country in handling and suppressing the COVID-19 outbreak, then the high number of daily confirmed cases reported in Indonesia indicates the poor implementation of the policy and government supervision of the LSSR. According to The Institute for Development of Economics and Finance [13], Indonesia is the least successful country in implementing the movement restriction policy among Southeast Asian countries. The number of confirmed cases in Indonesia is considered the worst compared with neighboring countries.

LSSR implementation in Indonesia indeed has faced many obstacles since its first inception. The success of LSSR is not merely determined by policy execution by the government, but is significantly influenced by public willingness to participate by voluntarily staying at home and not visiting public places. Therefore, it is useful to examine public response toward the implementation of LSSR. However, studies on public response toward LSSR effectiveness are limited. Most prior public-oriented studies have focused on COVID-19 knowledge, attitudes, and behaviors [14-19]. Furthermore, only few studies discussing public response toward social quarantine policy, especially in Indonesia, are available.

Accordingly, this study aims to identify public response toward government policy, specifically the LSSR, in Indonesia. Studies on public response toward policy implementation regarding COVID-19 prevention are imperative, particularly when the government as the policy maker is expected to be sensitive to societal needs and behavior [20]. Utilizing sentiment analysis, this study attempts to identify Indonesian public sentiment and emotions toward the LSSR from a macro perspective. Several studies have examined public response toward government rules concerning COVID-19 prevention in Indonesia using sentiment analysis, for example, Djalante et al [11] and Raamkumar et al in Singapore [21]. However, both studies looked at responses on specific platforms and cannot be generalized to the larger population. Therefore, this study will offer a macro approach by mining and analyzing big data from more than 500 online news portals and social media platforms such as Twitter, Facebook, Instagram, and YouTube. This study is useful to gauge public responses in Indonesia in detail as guidance to improve existing policy.

## Methods

### Study Design

**Figure 1:**
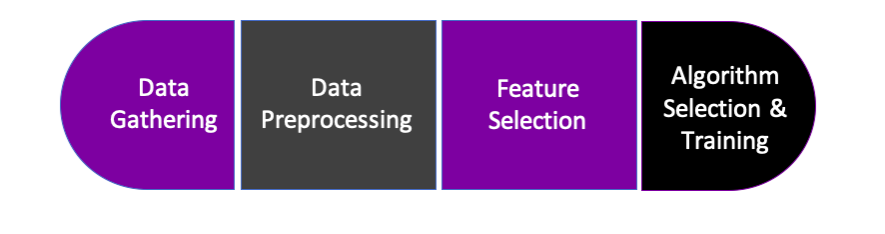
Machine learning workflow.

This study employed sentiment analysis using a supervised machine learning approach to mine COVID-19-related posts on selected media platforms (Twitter, Facebook, Instagram, and YouTube). Machine learning was chosen as the method for classifying sentiment categories considering its effectiveness to accommodate a larger data set. The analysis was also performed on COVID-19 news published in more than 500 online news platforms recognized by the Indonesian Press Council. Online news and social media posts included in the analysis are those posted between March 31 and May 31, 2020. The whole process of supervised machine learning application is described in Figure 1.

The first analysis in this study involved sentiment analysis on data from both online news portals and social media platforms. The second analysis was an emotion analysis conducted on the Twitter platform to identify collective public emotion toward the LSSR. The whole analysis process on social media and online news portal involved natural language processing (NLP) on Apache SOLR. Details of the systematic analysis process are as outlined below.

### Data Gathering

This study applied 2 different methods for data collection from each platform. The data from online news portals were collected using Perl Web Crawler, while real-time application programming interface (API) provided by Perl Programming Language, including Net::Twitter, Facebook::Graph, API::Instagram, and WebService::YouTube, was employed for each social media platform. Online news and social media posts obtained were then stored in the Apache SOLR database. Search profiling was accomplished using the terms “*Pembatasan Sosial Berskala Besar*” (large-scale social restrictions), “*Pembatasan Sosial*” (Social Restrictions), and “*PSBB*” (LSSR) as the main keywords to describe the government-implemented social restrictions in Indonesia. These keywords were deemed sufficient to capture public interest in the LSSR as there are no other synonyms in either Bahasa Indonesia or English that refer specifically to the government-implemented restrictions.

### Data Preprocessing

#### Overview

Additionally, a data cleansing process was conducted. The supervised machine learning approach enabled the researchers to sort, select, and also update the data stored in the search engine to ensure that only data associated with the study context will be analyzed. The main purpose of data preprocessing is to clean the data set from all noise and outliers. The details of this phase are described in the following subsections.

#### Duplicate Text Filtering

This process was done to remove text duplication and ensure only original data were included for analysis. All duplications in social media data (mostly from retweets) and online news were removed to avoid redundancy.

#### Text Normalization

All usernames, stop words (eg, at, in, on, may, always), URLs, hash symbols (#), punctuation marks, and other nonalphanumeric characters were removed as those entities would have no influence in determining the value of sentiment. Besides, typos and multiple occurrences of certain characters within 1 word were normalized in this stage (eg, “SAAAAAAAAAD” becomes “SAD”).

#### Case Folding

Case folding is a procedure that helps data normalization, in which all texts are standardized to lower case to make it easier for the system to retrieve information more effectively.

### Feature Selection (Part-of-Speech Tagging)

The part-of-speech (POS) tagging system is a word processing tool in NLP that refers to the process of tagging text onto a corresponding POS based on its definition and its relation with other words [22]. Commonly, only words that are categorized as nouns, adjectives, verbs, and adverbs were tokenized and tagged by the POS tagger, as those words are considered important indicators of objectivities and opinions [23]. Some studies have been conducted to create an Indonesian Standardized Annotated Corpus by applying a probability statistic approach such as Hidden Markov Model [24] and Maximum Entropy [25]; nevertheless, none of them are accessible for general audiences [26]. Thus, in this study, it was decided that the Indonesian Language Dictionary (*Kamus Besar Bahasa Indonesia* or *KBBI*) would be used as the main lexical database.

### Algorithm Selection and Training

The naïve Bayes classifier is a machine learning algorithm that is commonly utilized for text classification based on probabilistic calculations to predict future possibilities by looking at past patterns [27]. Although there is still no exact justification on which algorithm is best in classifying text, the use of naïve Bayes has been proven effective or even more accurate [28] and precise [23,29] than other algorithms in previous studies. Additionally, a recent study discovered naïve Bayes to be more accurate in determining COVID-19 sentiment categories compared with other classifiers such as logistic regression [30]. Therefore, it was decided that this algorithm will be used to classify the sentiment into 3 categories from the training data: positive, negative, and neutral. The use of naïve Bayes algorithm for this study can be described by the following equation:


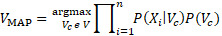


where *V*_MAP_ is all examined categories; *V_c_* is the sentiment category; *P*(*X_i_*|*V_c_*) is the probability of the word *i* falling into category *c*; and *P*(*V_c_*) is the probability of *V_c_*.

Large amounts of data tokenized into nouns, adjectives, verbs, and adverbs by POS tagging were stored within a training data set and trained using fivefold cross validation to ensure the accuracy of the sentiments. To further improve the accuracy, a system that allows admin intervention in the sentiment labeling process was developed and interannotator agreement was applied involving 3 annotators (from Astramaya) in the review process to ensure the validity of labeling.

In the review process, all unrelated contents were removed as they are not suitable for analysis. Further, this process enabled the researchers to fix the sentiment defined on the machine so that the sentiment tendency would fit to the context and demands of the analysis. After the selection process, we obtained 1,934,575 mentions of the search terms on both online news portals and social media platforms from March 31 to May 31, 2020. The data crawling process placed Twitter as the platform with the highest number of data, in which the keywords were mentioned 1,440,062 times. The use of Twitter for sentiment analysis research is indeed more popular than other platforms. Twitter is accessed by over 330 million users monthly and has 145 million daily active users [31]. The open nature of the microblogging service with numerous daily messages produced and generated has positioned it as the focal point of social media research and NLP [32].

### Lexicon-Based Approach for Emotion Analysis

The emotion analysis in this study was performed using a lexicon-based, or specifically a dictionary-based approach, in which the Indonesian Language dictionary *Kamus Besar Bahasa Indonesia* (*KBBI*) was utilized as the lexical database. The categorization of emotion was derived from the basic emotion concept put forth by Plutchik [33], including joy, trust, fear, surprise, sadness, disgust, anger, and anticipation. There is a slight methodology difference in determining emotion categories for the data collected from both types of platforms. On social media, public sentiment and emotion were identified based on an analysis of the text in each post; 1 post or unit of analysis could represent 1 or more emotions. Emotion analysis with a lexicon-based approach enables the researcher to conduct pattern matching using the regular expression feature on the Perl application. In addition, emotion analysis was not carried out for online news platform as online news does not represent any form of public conversation trend. Thus, only public sentiment can be obtained on this platform, and not public emotion.

### Data Visualization

Visualization is the final part of the NLP process, where the results of the analysis are translated into visuals to support the statistical data. To optimize the data presentation interface, all visual presentations in this study were delivered using Zend Framework (Linux Foundation).

## Results

### Data Demography

**Figure 2:**
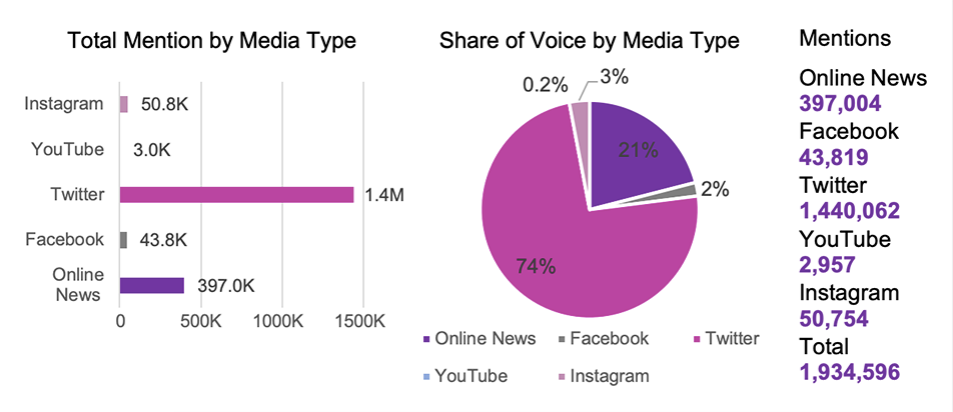
Data distribution for each platform.

Search using the keywords “*Pembatasan Sosial*” and “*PSBB*” (LSSR) performed on both online news and social media platforms revealed 1,934,596 mentions of these in news sentences and social media posts. Twitter yielded the highest mentions (1,440,062/1,934,596, 74.44%), followed by online news (397,004/1,934,596, 20.52%), Instagram (50,754/1,934,596, 2.62%), Facebook (43,819/1,934,596, 2.27%), and YouTube (2957/1,934,596, 0.15%). Figure 2 presents the data distribution for each platform.

**Figure 3:**
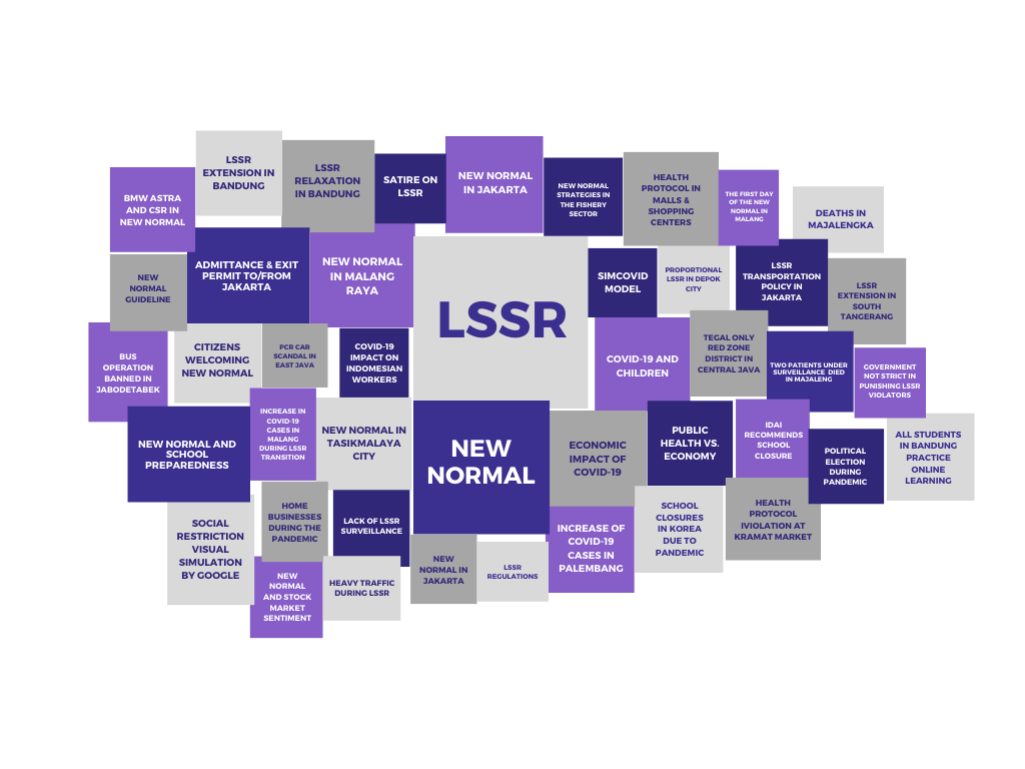
Topic mapping of online news platforms.

The data distribution showed that discussions related to LSSR on social media mainly took place on Twitter. This justified the use of Twitter to describe the overall sentiment and emotions toward the LSSR on social media. To identify popular topics discussed by the public, the NLP process helped to cluster discourses which were predominantly used and written in online news platforms via topic mapping. However, on Twitter, popular topics were identified based on top tweets during the study period. Figure 3 reports discourses and topic mapping for LSSR in online news platforms.

The most frequently discussed topics related to LSSR in Indonesia are LSSR policy, the new normal, and the impact of LSSR on the economic sector. The high level of conversation regarding the relaxation of LSSR in online news platforms represents the amplification of various responses from the public and media toward the government’s swift plan to implement new norms.

The discussions about LSSR on both online news platforms and social media are mostly in relation to the new normal. In addition, news crawling found that a number of LSSR-related news are political. Many assumed that the LSSR relaxation in Indonesia was not based on careful consideration but was implemented to reduce the economic burden on the government. The term “political crisis” was used to describe what would happen if the government did not take any immediate action to ease the economic burden of its society. This assumption is supported by the high number of new reports highlighting the economic impact of LSSR on the nation. Government as the policy maker faced various negative reverberations about LSSR relaxation, as it was seen to be prioritizing the economy over public health.

**Figure 4:**
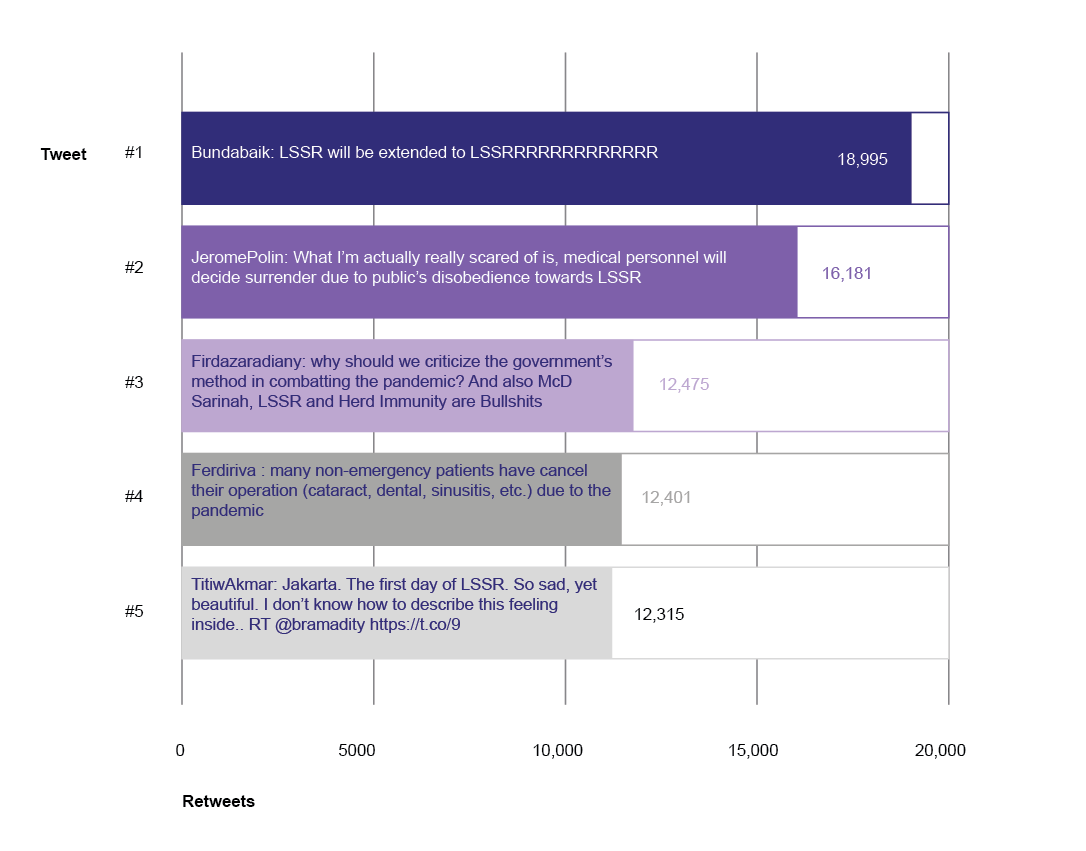
Most retweeted tweets.

In addition, to identify LSSR topics with the highest engagement level on Twitter, tweets with the highest number of retweets were ranked. Figure 4 describes 5 tweets with the highest engagement.

Overall, it can be assumed that the tweets represented public outburst and resentment toward 2 parties: the government that is considered indecisive to implement LSSR, and those among the public who enjoyed the LSSR relaxation by going out freely.

### Public Sentiment Toward Large-Scale Social Restrictions

**Figure 5:**
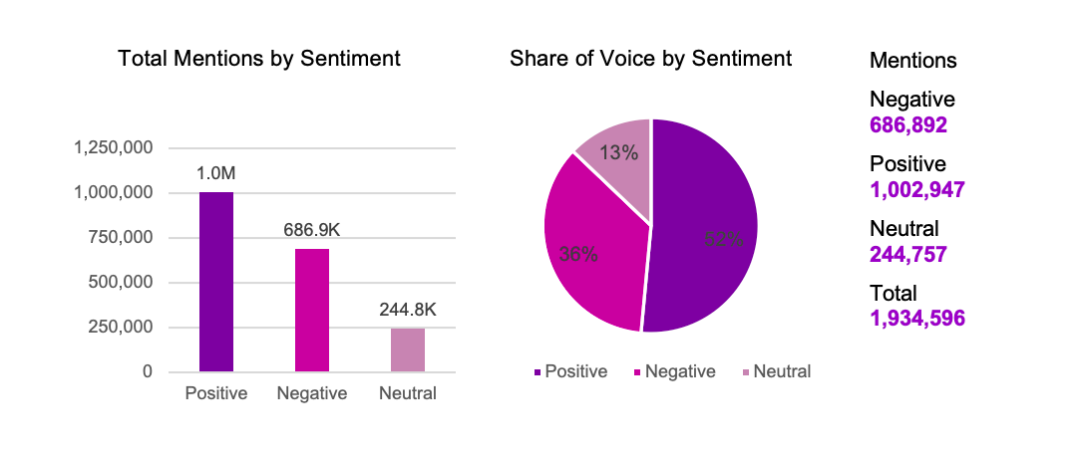
Total mentions by sentiment.

Sentiment analysis on online news platforms and social media during the period between March 31 and May 31, 2020 (n=1,934,596) showed that public sentiment toward the LSSR tended to be positive. Figure 5 presents the distribution of total mentions of positive, negative, and neutral sentiments in online news and social media platforms.

**Figure 6:**
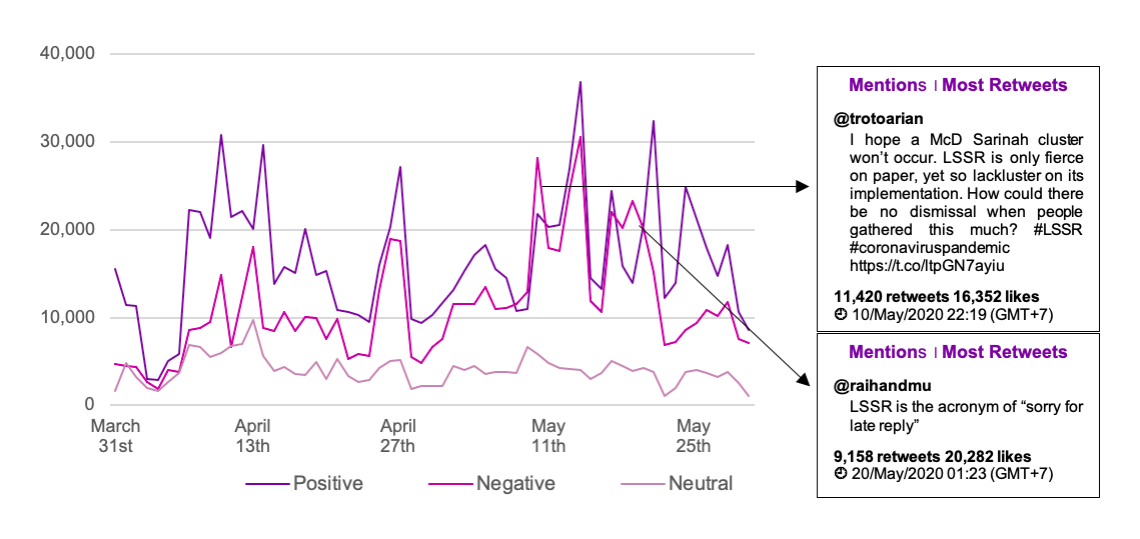
Sentiment trends in all media types.

Based on the statistics in Figure 5, positive sentiment surpasses other sentiment categories (1,002,947/1,934,596, 51.84%) followed by negative (686,892/1,934,596, 35.51%) and neutral (244,757/1,934,596, 12.65%) sentiments. These data suggest that although society in general viewed the government’s LSSR implementation and relaxation as indecisive, the majority still supported and regarded LSSR as the best solution to break the chain of COVID-19 transmission in the country. At the same time the negative sentiment on LSSR can be considered very high. It is not a good indicator of government performance, moreover, considering that the recovery phase of this health crisis will highly depend on public support and cooperation. Therefore, strategic and proactive efforts are needed to maximize the support from society. To deepen the understanding of public sentiment, a trend analysis was carried out as outlined in Figure 6.

Figure 6 presents the overall trend in sentiment for both online news and social media. It was observed that overall, positive sentiments were higher than negative and neutral sentiments. There were only 2 days where negative sentiments were higher than positive sentiments, precisely on May 11 and 21, 2020. Tweets with the highest engagement on May 11, 2020, highlighted the flaws in LSSR implementation, wherein the government lacked a firm response toward a mass gathering in Sarinah, Central Jakarta.

**Figure 7:**
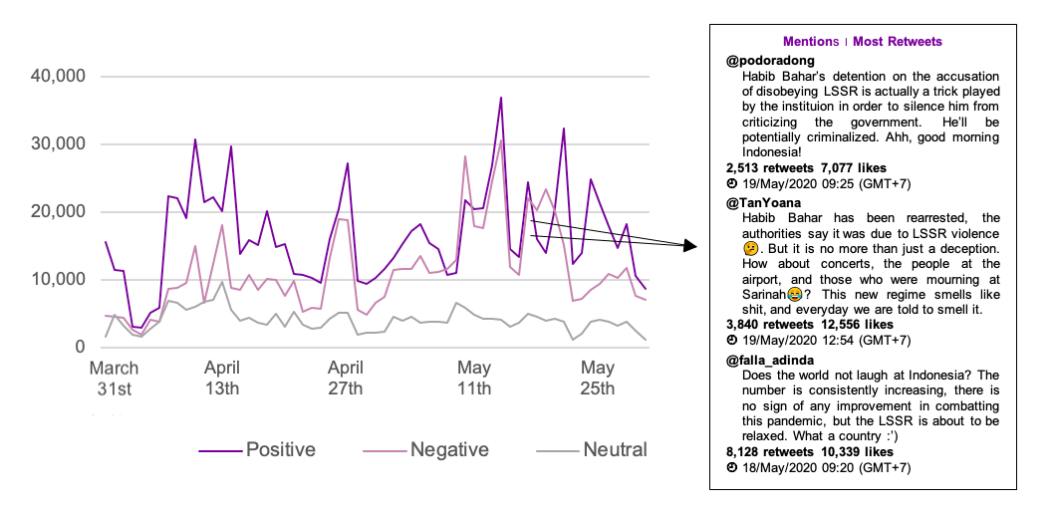
Sentiment trends on May 17, 2020.

The positive and negative sentiments toward the LSSR reached the peak on the same day (ie, May 14, 2020), with recorded mentions of more than 30,000. Interestingly, both positive and negative opinions on that day were referring to a particular tweet, posted by @Jeromepolin, regretting the public’s noncompliance to the LSSR. A detailed analysis concerning the discussion trend revealed that the negative sentiments referred mostly to the indecisive government and noncompliance toward the LSSR. A similar pattern of both positive and negative sentiments with high mentions was also recorded on May 17, 2020 (Figure 7).

Similar to the sentiment trends on May 14, 2020, the topic with the highest mentions on May 17, 2020, also referred to skepticism toward the government’s implementation of the LSSR. In addition to this, some parties were disappointed with the government’s decision to relax the LSSR when positive cases of COVID-19 in Indonesia were still on the rise.

### Public Emotions Toward LSSR

**Figure 8:**
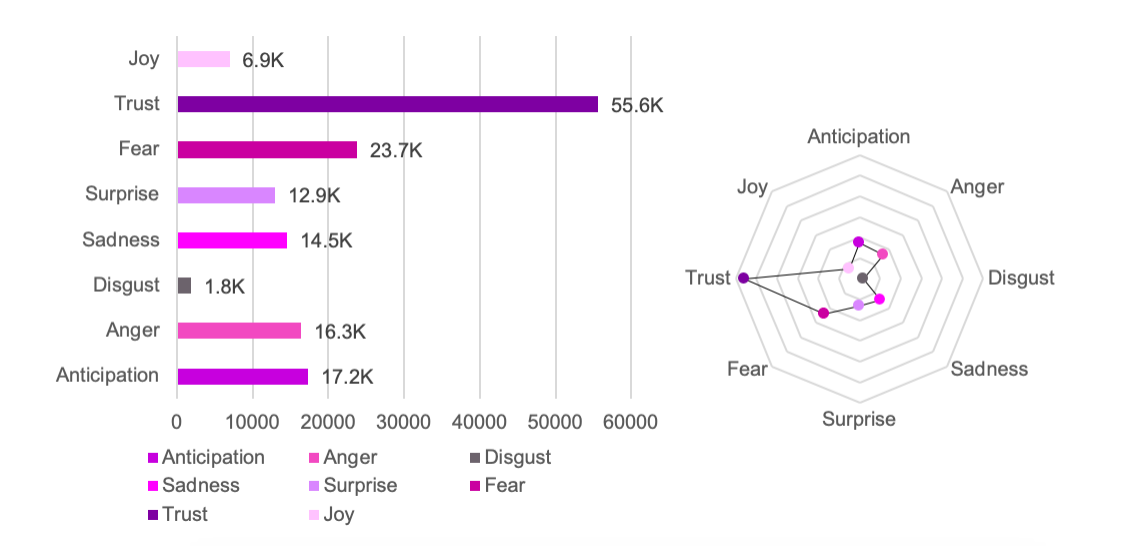
Emotion analysis.

Public emotion analysis using a lexicon-based approach and quote extraction was applied to explore collective public emotion regarding the LSSR. As discussed in the “Methods” section, public emotion was categorized based on the basic emotion concept by Plutchik [33], including joy, trust, surprise, fear, disgust, sadness, anger, and anticipation. Furthermore, this analysis can be used as a parameter to identify how far the government and the LSSR garnered public trust. Public emotions toward the LSSR are shown in Figure 8.

**Figure 9:**
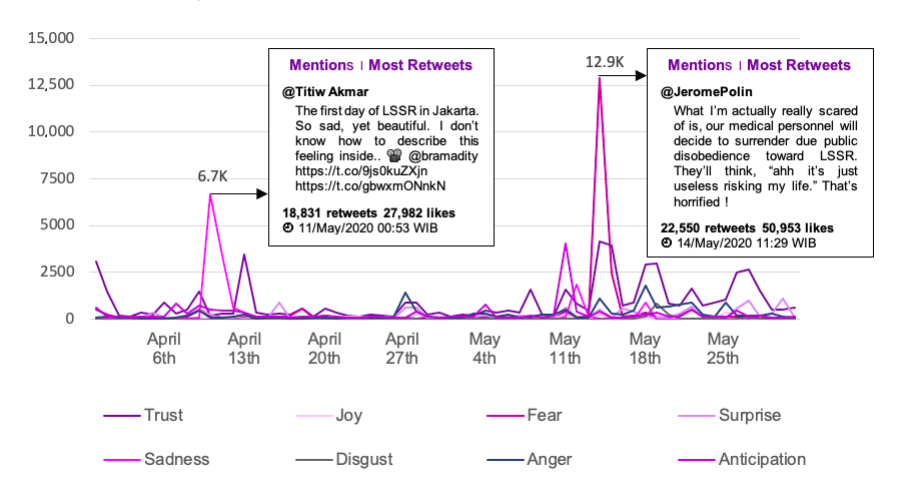
Trend of public emotions.

The emotion analysis concluded that although there were many negative opinions toward the government and the LSSR, the public still believed that the LSSR was the best method to break the chain of COVID-19 transmission. The public expected that all members of society would accept and comply with this policy in order to achieve overall success. At the same time, the public still had expectations that the government would immediately overcome the issue by implementing firmer control. Although the overall public emotion was still “trustful,” interesting trends were found in 2 other emotion categories, namely, “fear” and “sadness.” Figure 9 depicts public emotion trends during the study period.

The “sadness” and “fear” emotions showed the most outstanding patterns compared with other emotion categories and were found to reach their peak on April 11, 2020, and May 14, 2020, respectively. On April 11, 2020, the most retweeted post suggested public sadness toward the implications of the LSSR. Moreover, on that date, the number of deaths continued to surpass the total recovered cases in the country. The same trend occurred on 22 May, 2020, which also indicated “fear” of public disobedience toward the LSSR.

## Discussion

### Principal Findings

This study found Twitter as the most dominant platform used by the public when discussing COVID-19 and LSSR. Public sentiment toward the LSSR was largely positive with expressions of support for the government intervention in combating COVID-19. Even so, negative sentiments were also quite high, especially concerning government’s indecisiveness and public defiance of the LSSR. The emotion analysis revealed that Indonesians were mostly trustful with notable peaks in sadness and fear corresponding to increased COVID-19 death rates.

A closer examination of tweets with the highest level of engagement showed that a majority of the public were dissatisfied with the government and parties that did not obey LSSR regulations. This is supported by the trends observed in the sentiment analysis. Criticisms of government’s indecisiveness and LSSR relaxation dominated the discussion trends regarding LSSR during the study period. Even though the overall public sentiment was positive, the negative and neutral sentiments were still considered high. The fact that almost half of the public did not show positive attitudes toward the government and LSSR implementation can be worrying, particularly in a global health crisis of this magnitude. A neighboring country, Vietnam, for example, has proven that effective cooperation between the society and the government can successfully allow the country to control the spread of COVID-19 cases. Compliance of Vietnamese to their government instructions such as wearing masks and implementing self-quarantine [34] has made their country one of the victorious in managing the pandemic with less than 60 deaths as of June 2021 [35].

The results also show that the Indonesian government’s inconsistent stance in implementing policies has contributed to the emergence of public emotions such as fear and sadness. The Indonesian media may have played a role in this, as concerns about socioeconomic uncertainty, LSSR, social distancing violations, and economic downturn in all sectors were widely reported in the media from March to early April 2020 [36]. In the earlier phase of the pandemic, negative media reports on issues such as insufficient government response, patient care, and burial of the deceased also caused agitation among the Indonesian public [37]. A previous study on COVID-19 news in Indonesia also suggested that the distortion of news by both online and mainstream media has caused an increase in public fear and anxiety [38].

Analysis of sentiments surrounding COVID-19 has produced similar results in communities across the globe. The health crisis has sparked concerns on the spread of the virus and its containment [39], economic survival [40], and physical and mental well-being [41]. The uncertainty surrounding the pandemic has resulted in expressions of mixed emotions in the public domain. An analysis of Twitter discussions in the early stages of the pandemic found dominant emotions of trust, anger, and fear surrounding COVID-19 cases and deaths [39]. A study in Italy observed a similar pattern; feelings of anger and fear emerged along with trust, solidarity, and hope [42], while in Spain sentiments surrounding the pandemic were that of disgust, fear, anger, and sadness [43].

It is important to note that while these sentiments and emotions were dominant at the beginning of the pandemic in early 2020, there has been much polarity and change in the sentiments since then [44]. COVID-19 should be a lesson for governments to affirm the consequential role of strategic public policy and acknowledge the importance of clear, consistent, and well-targeted public communications in overcoming crisis.

While public trust is still high, the government must be responsive to the needs of its society, considering their outlook as the basis for formulating policies and revising those deemed incompatible with public needs. Economic struggles were one of the biggest drivers of LSSR noncompliance in Indonesia. The large number of violations found in big cities such as Jakarta, Bogor, Depok, Tangerang, and Bekasi [45] is consistent with the findings of Wasdani and Prasad [46], who reported that the implementation of social distancing among the urban poor is very difficult. Therefore, strategic public policy should be introduced to maintain economic resilience, for instance, allocating budget for economic stimulus packages as done by the Malaysian government, which distributed MYR 260 billion (US $61 billion) to its people in the lower- and medium-income groups [47].

Even so, robust public policy alone may not be sufficient to curb negative emotions surrounding the pandemic. Mohamad et al [48] have highlighted that the delivery of clear, consistent, and credible information is key to control and mitigate the disease. Misinformation resulting from the communication of policies greatly affects public perceptions and trust during the pandemic [49]. To ensure successful communication to the public, authorities must recognize the important role of mass media in delivering quality information, as evidenced by previous studies [43].

### Strength and Limitations

The strength of this study is in the variety of platforms analyzed. To obtain more diverse data, analyzing various types of social media and online news platforms with relatively large data sets makes it possible to synthesize diverse results compared with previous studies. Previous studies involving online media often only analyzed a single type of platform such as Twitter [49,50], Sina Weibo, the Chinese version of Twitter [51,52], Facebook [21], and online news portals [53,54]. By contrast, this study has a relatively larger data set collected by optimizing web-crawling functions on more than 500 online news portals and large amounts of social media data.

However, there were some limitations to this study. The large amount of data analyzed on social media platforms made it difficult for the researchers to determine what topics were dominant on social media, particularly Twitter. The text-based approach used on Twitter requires a relatively longer issue clustering process. Accordingly, the topic mapping could only be used to analyze the data gathered from online news platforms, by applying a clustering method to identify topics that are similar to each other, assisted by paragraph segmentation to deepen researcher understanding of the news context. In addition, this study did not conduct emotion analysis on the comments that appeared in online news. Future research is recommended to explore public emotions by analyzing comments written in response to news items.

### Research Implications

The results of this study may assist policy makers in being active observers of public responses in online media, especially on social media. Public communications by the government in this new media era must transform from being mere publicity to observing and analyzing public sentiment.

Social listening is becoming an important way to gauge public opinion and response. Public confusion surrounding the LSSR is a sign that the government must develop a comprehensive communication protocol to emphasize the importance of LSSR in eradicating the spread of COVID-19 in Indonesia.

### Conclusions

Overall, this study disclosed Twitter as the most popular platform used by Indonesian public in conveying their thoughts regarding LSSRs. The analysis performed on the major keywords that appeared on social media and online news portals revealed positive sentiment toward the LSSR. However, there were concerns on emerging conversations regarding public skepticism toward effective implementation of the LSSR. In addition, the emotion analysis concluded that a majority of the public still believe LSSR as the best method to effectively slow the spread of COVID-19. This explains why the public still hold high hopes and expectations toward the government as the policy maker in keeping them safe in a health crisis such as this.

## Abbreviations

API: application programming interface
*KBBI*: *Kamus Besar Bahasa Indonesia*
LSSR: large-scale social restrictions
NLP: natural language processing
POS: part-of-speech

## References

[1] Askitas N, Tatsiramos K, Verheyden B (2021). Estimating worldwide effects of non-pharmaceutical interventions on COVID-19 incidence and population mobility patterns using a multiple-event study. Sci Rep.

[2] Mehta RM, Mehta R, Balaji AL, Mehta H (2020). Perceptions on COVID19: A ground-level analysis to guide public policy. Lung India.

[3] Punn N, Sonbhadra S, Agarwal S (2021). Monitoring Covid-19 Social Distancing with Person Detection and Tracking via Fine-tuned YOLO v3 and Deepsort Techniques. arXiv.

[4] Jarvis CI, Van Zandvoort K, Gimma A, Prem K, Klepac P, Rubin GJ, Edmunds WJ, CMMID COVID-19 working group (2020). Quantifying the impact of physical distance measures on the transmission of COVID-19 in the UK. BMC Med.

[5] Vogel L (2020). How long will social distancing take to work? Experts weigh in on Canada's COVID-19 response. CMAJ.

[6] Wong J, Koh WC, Alikhan MF, Abdul Aziz ABZ, Naing L (2020). Responding to COVID-19 in Brunei Darussalam: Lessons for small countries. J Glob Health.

[7] Hsieh C, Lin C, Wang WYC, Pauleen DJ, Chen JV (2020). The Outcome and Implications of Public Precautionary Measures in Taiwan-Declining Respiratory Disease Cases in the COVID-19 Pandemic. Int J Environ Res Public Health.

[8] Ng CFS, Seposo XT, Moi ML, Tajudin MABA, Madaniyazi L, Sahani M (2020). Characteristics of COVID-19 epidemic and control measures to curb transmission in Malaysia. Int J Infect Dis.

[9] Abideen AZ, Mohamad FB, Hassan MR (2020). Mitigation strategies to fight the COVID-19 pandemic—present, future and beyond. JHR.

[10] Setiati S, Azwar MK (2020). COVID-19 and Indonesia. Acta Med Indones.

[11] Djalante R, Lassa J, Setiamarga D, Sudjatma A, Indrawan M, Haryanto B, Mahfud C, Sinapoy MS, Djalante S, Rafliana I, Gunawan LA, Surtiari GAK, Warsilah H (2020). Review and analysis of current responses to COVID-19 in Indonesia: Period of January to March 2020. Prog Disaster Sci.

[12] Channel News Asia Channel News Asia.

[13] INDEF: Indonesia's Large-Scale Social Restrictions Is The Worst Among Neighboring Country. Detik News.

[14] McFadden SM, Malik AA, Aguolu OG, Willebrand KS, Omer SB (2020). Perceptions of the adult US population regarding the novel coronavirus outbreak. PLoS One.

[15] Mahmood S, Hussain T, Mahmood F, Ahmad M, Majeed A, Beg BM, Areej S (2020). Attitude, Perception, and Knowledge of COVID-19 Among General Public in Pakistan. Front Public Health.

[16] Azlan AA, Hamzah MR, Sern TJ, Ayub SH, Mohamad E (2020). Public knowledge, attitudes and practices towards COVID-19: A cross-sectional study in Malaysia. PLoS One.

[17] Maheshwari S, Gupta P, Sinha R, Rawat P (2020). Knowledge, attitude, and practice towards coronavirus disease 2019 (COVID-19) among medical students: A cross-sectional study. J Acute Dis.

[18] Khasawneh AI, Humeidan AA, Alsulaiman JW, Bloukh S, Ramadan M, Al-Shatanawi TN, Awad HH, Hijazi WY, Al-Kammash KR, Obeidat N, Saleh T, Kheirallah KA (2020). Medical Students and COVID-19: Knowledge, Attitudes, and Precautionary Measures. A Descriptive Study From Jordan. Front Public Health.

[19] Al-Hanawi MK, Angawi K, Alshareef N, Qattan AMN, Helmy HZ, Abudawood Y, Alqurashi M, Kattan WM, Kadasah NA, Chirwa GC, Alsharqi O (2020). Knowledge, Attitude and Practice Toward COVID-19 Among the Public in the Kingdom of Saudi Arabia: A Cross-Sectional Study. Front Public Health.

[20] Waitzberg R, Davidovitch N, Leibner G, Penn N, Brammli-Greenberg S (2020). Israel's response to the COVID-19 pandemic: tailoring measures for vulnerable cultural minority populations. Int J Equity Health.

[21] Sesagiri Raamkumar A, Tan SG, Wee HL (2020). Measuring the Outreach Efforts of Public Health Authorities and the Public Response on Facebook During the COVID-19 Pandemic in Early 2020: Cross-Country Comparison. J Med Internet Res.

[22] Alfred R, Mujat A, Obit JH (2013). A ruled-based part of speech (RPOS) tagger for Malay text articles.

[23] Vidya NA, Budi I, Fanany MI (2015). Twitter sentiment to analyze net brand reputation of mobile phone providers.

[24] Wicaksono AF, Purwarianti A (2010). HMM based part-of-speech tagger for Bahasa Indonesia. https://www.researchgate.net/publication/209387036_HMM_Based_Part-of-Speech_Tagger_for_Bahasa_Indonesia.

[25] Nurwidyantoro A, Winarko E (2012). Parallelization of maximum entropy POS tagging for Bahasa Indonesia with MapReduce. International Journal of Computer Science Issues.

[26] Muljono M, Afini U, Supriyanto C, Nugroho RA (2017). The Development of Indonesian POS Tagging System for Computer-aided Independent Language Learning. Int. J. Emerg. Technol. Learn.

[27] Rozi IF, Hamdana EN, Iqbal Alfahmi MB (2018). Development of twitter sentiment analysis using naïve bayes classifier method (case study of SAMSAT in Malang city). Jurnal Informatika Polinema.

[28] Sailunaz K, Alhajj R (2019). Emotion and sentiment analysis from Twitter text. Journal of Computational Science.

[29] Le B, Nguyen N, Le Thi H, Nguyen N, Do T (2015). Twitter sentiment analysis using machine learning techniques. Advanced Computational Methods for Knowledge Engineering. Advances in Intelligent Systems and Computing.

[30] Samuel J, Ali GGMN, Rahman MM, Esawi E, Samuel Y (2020). COVID-19 public sentiment insights and machine learning for Tweets classification. Information.

[31] Lin Y (2021). 10 Twitter Statistics Every Marketer Should Know in 2020 [Infographic]. Obelro.

[32] Stojanovski D, Strezoski G, Madjarov G, Dimitrovski I, Chorbev I (2018). Deep neural network architecture for sentiment analysis and emotion identification of Twitter messages. Multimed Tools Appl.

[33] Plutchik R (2001). The Nature of Emotions. Am. Sci.

[34] La V, Pham T, Ho M, Nguyen M, P. Nguyen K, Vuong T, Nguyen HT, Tran T, Khuc Q, Ho M, Vuong Q (2020). Policy Response, Social Media and Science Journalism for the Sustainability of the Public Health System Amid the COVID-19 Outbreak: The Vietnam Lessons. Sustainability.

[35] Worldometer.

[36] Gandasari D, Dwidienawati D (2020). Content analysis of social and economic issues in Indonesia during the COVID-19 pandemic. Heliyon.

[37] Widati S, Soedirham O, Wee EH (2021). Social construction of COVID-19 destigmatization through Indonesia's online mass media. International Journal of Current Research.

[38] Sulistiadi W, Rahayu S, Harmani N (2020). Handling of Public Stigma on COVID-19 in Indonesian Society. Jurnal Kesehatan Masyarakat Nasional (National Public Health Journal).

[39] Xue J, Chen J, Hu R, Chen C, Zheng C, Su Y, Zhu T (2020). Twitter Discussions and Emotions About the COVID-19 Pandemic: Machine Learning Approach. J Med Internet Res.

[40] El Keshky MES, Basyouni SS, Al Sabban AM (2020). Getting Through COVID-19: The Pandemic's Impact on the Psychology of Sustainability, Quality of Life, and the Global Economy - A Systematic Review. Front Psychol.

[41] Wang C, Tee M, Roy AE, Fardin MA, Srichokchatchawan W, Habib HA, Tran BX, Hussain S, Hoang MT, Le XT, Ma W, Pham HQ, Shirazi M, Taneepanichskul N, Tan Y, Tee C, Xu L, Xu Z, Vu GT, Zhou D, Koh BJ, McIntyre RS, Ho C, Ho RC, Kuruchittham V (2021). The impact of COVID-19 pandemic on physical and mental health of Asians: A study of seven middle-income countries in Asia. PLoS One.

[42] Stella M, Restocchi V, De Deyne S (2020). #lockdown: Network-Enhanced Emotional Profiling in the Time of COVID-19. BDCC.

[43] de Las Heras-Pedrosa C, Sánchez-Núñez Pablo, Peláez José Ignacio (2020). Sentiment Analysis and Emotion Understanding during the COVID-19 Pandemic in Spain and Its Impact on Digital Ecosystems. Int J Environ Res Public Health.

[44] Wicke P, Bolognesi MM (2021). Covid-19 Discourse on Twitter: How the Topics, Sentiments, Subjectivity, and Figurative Frames Changed Over Time. Front. Commun.

[45] Taufan SA Large-scale Social Restrictions Violation in Jadetabek has reached 70 thousand cases. Jawa Pos.

[46] Wasdani KP, Prasad A (2020). The impossibility of social distancing among the urban poor: the case of an Indian slum in the times of COVID-19. Local Environment.

[47] Aziz NA, Othman J, Lugova H, Suleiman A (2020). Malaysia's approach in handling COVID-19 onslaught: Report on the Movement Control Order (MCO) and targeted screening to reduce community infection rate and impact on public health and economy. J Infect Public Health.

[48] Mohamad E, Tham JS, Ayub SH, Hamzah MR, Hashim H, Azlan AA (2020). Relationship Between COVID-19 Information Sources and Attitudes in Battling the Pandemic Among the Malaysian Public: Cross-Sectional Survey Study. J Med Internet Res.

[49] Sell TK, Hosangadi D, Trotochaud M (2020). Misinformation and the US Ebola communication crisis: analyzing the veracity and content of social media messages related to a fear-inducing infectious disease outbreak. BMC Public Health.

[50] Abd-Alrazaq A, Alhuwail D, Househ M, Hamdi M, Shah Z (2020). Top Concerns of Tweeters During the COVID-19 Pandemic: Infoveillance Study. J Med Internet Res.

[51] Liao Q, Yuan J, Dong M, Yang L, Fielding R, Lam WWT (2020). Public Engagement and Government Responsiveness in the Communications About COVID-19 During the Early Epidemic Stage in China: Infodemiology Study on Social Media Data. J Med Internet Res.

[52] Zhao Y, Cheng S, Yu X, Xu H (2020). Chinese Public's Attention to the COVID-19 Epidemic on Social Media: Observational Descriptive Study. J Med Internet Res.

[53] Liu Q, Zheng Z, Zheng J, Chen Q, Liu G, Chen S, Chu B, Zhu H, Akinwunmi B, Huang J, Zhang CJP, Ming W (2020). Health Communication Through News Media During the Early Stage of the COVID-19 Outbreak in China: Digital Topic Modeling Approach. J Med Internet Res.

[54] Rovetta A, Bhagavathula AS (2020). COVID-19-Related Web Search Behaviors and Infodemic Attitudes in Italy: Infodemiological Study. JMIR Public Health Surveill.

